# IL-33 Mediates Lung Inflammation by the IL-6-Type Cytokine Oncostatin M

**DOI:** 10.1155/2020/4087315

**Published:** 2020-11-28

**Authors:** Fernando Botelho, Anisha Dubey, Ehab A. Ayaub, Rex Park, Ashley Yip, Allison Humbles, Roland Kolbeck, Carl D. Richards

**Affiliations:** ^1^McMaster Immunology Research Centre, Department of Pathology and Molecular Medicine, McMaster University, Hamilton, ON, Canada L8N 3Z5; ^2^Division of Pulmonary and Critical Care Medicine, Brigham and Women's Hospital, Harvard Medical School, Boston, MA 02115, USA; ^3^Early RIA, BioPharmaceuticals R&D, AstraZeneca, Cambridge CB4 0WG, UK; ^4^Early RIA, BioPharmaceuticals R&D, AstraZeneca, Gaithersburg, MD 20878, USA

## Abstract

The interleukin-1 family member IL-33 participates in both innate and adaptive T helper-2 immune cell responses in models of lung disease. The IL-6-type cytokine Oncostatin M (OSM) elevates lung inflammation, Th2-skewed cytokines, alternatively activated (M2) macrophages, and eosinophils in C57Bl/6 mice in vivo. Since OSM induces IL-33 expression, we here test the IL-33 function in OSM-mediated lung inflammation using IL-33-/- mice. Adenoviral OSM (AdOSM) markedly induced IL-33 mRNA and protein levels in wild-type animals while IL-33 was undetectable in IL-33-/- animals. AdOSM treatment showed recruitment of neutrophils, eosinophils, and elevated inflammatory chemokines (KC, eotaxin-1, MIP1a, and MIP1b), Th2 cytokines (IL-4/IL-5), and arginase-1 (M2 macrophage marker) whereas these responses were markedly diminished in IL-33-/- mice. AdOSM-induced IL-33 was unaffected by IL-6-/- deficiency. AdOSM also induced IL-33R+ ILC2 cells in the lung, while IL-6 (AdIL-6) overexpression did not. Flow-sorted ILC2 responded *in vitro* to IL-33 (but not OSM or IL-6 stimulation). Matrix remodelling genes col3A1, MMP-13, and TIMP-1 were also decreased in IL-33-/- mice. In vitro, IL-33 upregulated expression of OSM in the RAW264.7 macrophage cell line and in bone marrow-derived macrophages. Taken together, IL-33 is a critical mediator of OSM-driven, Th2-skewed, and M2-like responses in mouse lung inflammation and contributes in part through activation of ILC2 cells.

## 1. Introduction

The interleukin-1-type cytokine IL-33 acts as an “alarmin” or damage signal that facilitates the recruitment of inflammatory cells in T helper-2 (Th2) immune cell models of lung disease [1]. IL-33 lacks an N-terminal secretory peptide sequence, and similar to other alarmins, IL-33 is released from necrotic cells following tissue injury. Soluble IL-33 binds a receptor complex of two subunits: ST2 (also known as T1/ST2 or IL1RL) and IL-1RAcP [2]. This IL-33 receptor (IL-33R) complex has been shown to stimulate NF-*κ*B and MAP kinase cell signaling, and soluble forms of the IL-33 receptor ST2 subunit have been shown to inhibit IL-33 receptor signaling [3]. Binding of extracellular soluble IL-33 to IL-33 receptors on T helper type-2 (Th2) cells and type-2 innate lymphoid cells (ILC2 cells) is important for driving Th2 responses, such as those observed in parasitic infections, allergy, and asthma [4, 5]. Transgenic overexpression of soluble IL-33 in mice results in lethal inflammation and autoimmunity [6], and elevated levels of IL-33 are found in mouse models of allergic airway inflammation and in severe asthma in human patients [7, 8]. Moreover, single nucleotide polymorphisms in IL-33 have been observed in human asthma patients, and increases in IL-33 and IL-33 receptor expression (ST2) occur in individuals with chronic obstructive pulmonary disease and obstructive sleep apnea (reviewed in [9]). In the lung, airway smooth muscle cells and type 2 alveolar epithelial cells show expression of IL-33 [4, 10].

Cytokine and growth factor studies have shown that TNF, IL-1, or GM-CSF can regulate expression of IL-33, while IFN*γ* suppresses IL-33 levels [10–13]. We have recently shown that the gp130/IL-6-family cytokine Oncostatin M (OSM) can upregulate the nuclear expression of IL-33 in alveolar epithelial cells in the mouse lung [14]. The OSM receptor is composed of a heterodimeric gp130-OSMR*β* complex [15] that is expressed by lung stromal cells, and among gp130 cytokines, OSM is a potent inducer of gp130 signaling that leads to expression of proinflammatory target genes and extracellular matrix remodelling [16, 17]. We and others have shown overexpression of OSM, or administration of OSM protein results in a Th2-like phenotype (eosinophilia and IL-4, IL-5, and IL-13 cytokine production) [18–20] with an associated Arg1+ alternatively activated macrophage accumulation in the C57Bl/6 mouse lung [21]. As IL-33 plays a prominent role in other Th2 responses, we here tested whether IL-33 function is required for OSM-induced effects in vivo. We assess wild-type and IL-33-/- mice responses to AdOSM, examine ILC2 induction by OSM and their responses in vitro, and use IL-6 overexpression as a comparator. The results show that IL-33 is required for OSM-induced inflammatory functions in the mouse lung.

## 2. Materials and Methods

### 2.1. Animals

Female C57Bl/6 wild-type and C57Bl/6 IL-6-/- mice (8-10 weeks old) were purchased from the Jackson Laboratory (JAX; Bar Harbor, Maine). Female C57Bl/6 IL-33-/- (IL-33-/- or IL-33KO) mice were provided by MedImmune LLC (Gaithersburg, MD). All mice were housed in standard conditions with food and water *ad libitum*. All procedures were approved by the Animals Research Ethics Board at McMaster.

### 2.2. Administration of Adenovirus Constructs and Sample Collection

Wild-type and IL-33-/- mice were administered 5 × 10^7^ pfu of replication-deficient AdDl70 or Ad-encoding OSM (AdOSM) through the endotracheal route of administration as previously published [19, 22]. Mice were euthanized after 7 or 14 days and bled, and alveolar lavage was performed as previously described [19, 22]. Alveolar lavage was centrifuged, and supernatants were stored for future analysis by ELISA. Cell pellets were resuspended, counted, and subjected to cytocentrifugation at 300 rpm for two minutes. Differential counts were determined after staining with Hema-3 (Fisher Scientific, Toronto, ON, Canada). Left lungs were perfused with 10% formalin and fixed for 48 hours subsequent to histological preparation and histochemical staining. The right lung was snap frozen and stored at -80°C for RNA and protein extraction as previously described [23]. The left lung lobe was excised, inflated/fixed with formaldehyde, and subsequently processed for histology.

### 2.3. Analysis of Protein (Immunoblotting)

20 *μ*g of protein was loaded on 12% SDS-PAGE gels. Following separation by SDS-PAGE, proteins were transferred to a nitrocellulose membrane and blocked for one hour at room temperature using LI-COR Odyssey blocking buffer (Mandel Scientific, Guelph, ON, Canada) and then probed using the following antibodies at 4°C overnight: actin (I-19, Santa Cruz Biotechnology), diluted 1 : 2000; mouse IL-33 (AF3626, R&D Systems, Oakville, ON, Canada, or NESSY-1, Abcam Inc., Toronto, ON, Canada), diluted 1 : 1000; phosphorylated STAT3 (Cell Signaling Technology, Whitby, ON, Canada), diluted 1 : 2000; and total STAT3 (Cell Signaling Technology), diluted 1 : 1000. Primary antibodies were detected using LI-COR anti-Rabbit or anti-Goat IRDye infrared secondary antibodies at 1 : 5000 or 1 : 10000 dilution, respectively, and imaged using the LI-COR Odyssey infrared scanner. Blots were stripped using LI-COR NewBlot Nitro Stripping Buffer, as per the manufacturer's instructions, and then blocked and reprobed for different proteins. For examining oxidation of IL-33, 20 *μ*g of lung cell extracts or 15 *μ*l of bronchoalveolar lavage fluid (BALF) was loaded onto 12% Bis-Tris NuPage gels (Thermo Fisher Scientific, Burlington, ON, Canada) with MOPS running buffer under reducing or nonreducing conditions as previously described [24]. Reduced samples contained 2% beta-mercaptoethanol. Densitometry analysis of immunoblot band intensity was performed using Image Studio Lite software (LI-COR). IL-33 protein bands were normalized to actin; pSTAT3 was normalized to total STAT3 (tSTAT). The R&D Systems AF3626 anti-IL-33 antibody was used to detect IL-33 in cell lysates unless specified otherwise. Cell lysates were compared relative to untreated control samples to quantify fold changes in the signal, and lung homogenate samples were compared relative to lung homogenate samples from mice treated with AdDl70.

### 2.4. ELISA, Multiplex-Array, and Arginase-1/Nitric Oxide Analysis

DuoSet ELISA kits were purchased from R&D Systems to measure protein levels of mouse OSM, mouse IL-6, mouse Eotaxin-2 and mouse TNFalpha in BALF samples stored at -80°C. Multiplex-array analysis of BALF samples was performed using a cytokine/chemokine 31-multiplex array (Eve Technologies, Calgary, Alberta, Canada) for the detection of mouse eotaxin-1, KC, MIP1a, MIP1b, VEGF, IL-4, IL-5, IL-13, IL-12p70, and IFN*γ*. Arginase-1 activity and nitric oxide production in whole cell lysates and supernatants, respectively, was measured as previously described [23]. Quantification was completed as per manufacturer's instructions.

### 2.5. RNA Analysis by Real-Time PCR, Nanostring, and Chromogenic In Situ Hybridization (CISH)

Lung tissues were homogenized in TRIzol (Thermo Fisher Scientific) and RNA extracted as previously described [25]. RNA was reverse transcribed using SuperScript IV (Thermo Fisher Scientific), and levels of viral or mouse endogenously derived OSM were assessed by real-time Q-PCR (TaqMan) using primers with FAM-5′ end-labeled fluorogenic probes for OSM. Left and right primer and TaqMan probe hybridization oligonucleotide sequences for endogenous mouse OSM are as follows: GGAGGGTCTTCAGGGAATG, ATTCTGCGG GTTCCCTTG, and ACGCAGCCGGAGACAGAG. Left/right primer and TaqMan probe hybridization oligonucleotide sequences for viral OSM are as follows: GGATACCATCGCTTCATGG, TCTAGCGGCCGCCTATCTC, and CTTCAGGGAATGGGACGAT. VIC-5′ end-labeled fluorogenic probes were used for 18S control RNA. All obtained gene expression assays came from Applied Biosystems, Thermo Fisher Scientific.

For real-time PCR, 10 ng reversed-transcribed RNA from whole lung RNA extractions was combined with Universal Master Mix (containing UNG; Thermo Fisher Scientific) and predesigned Applied Biosystems TaqMan Gene Expression Assay primers and used in Real-Time PCR reactions for assaying gene expression of mouse col1A1, col3A1, MMP-13, and TIMP-1. Alternatively, expression of these genes was assayed using Nanostring technology (NanoString Technologies, Seattle, WA).

For chromogenic *in situ* hybridization (CISH), formalin-fixed paraffin-embedded lung tissue sections were pretreated with heat and protease prior to hybridization with target oligo probes (Advanced Cell Diagnostics (ACD), Newark, CA, US) for mouse IL-33 and OSM. Detection was obtained using the RNAscope® 2.5 Duplex Assay Specific Kit, and RNA staining signal was identified as punctate dots as per ACD protocols.

### 2.6. Isolation of Lung Mononuclear Cells and Flow Cytometric Analysis

Lung mononuclear cell suspensions were generated by mechanical mincing and collagenase digestion as previously described [22]. Debris was removed by passage through a 45-micrometer screen size nylon mesh, and cells were resuspended in PBS containing 0.3% bovine serum albumin (BSA) (Thermo Fisher Scientific) or in RPMI supplemented with 10% fetal bovine serum (FBS) (Sigma-Aldrich, Oakville, ON, Canada), 1% L-glutamine, and 1% penicillin/streptomycin (Thermo Fisher Scientific). 1 × 10^6^ lung mononuclear cells were washed once with PBS/0.3% BSA and stained with primary antibodies directly conjugated to fluorochromes, for 30 minutes at 4°C. 10^5^ live events were acquired on an LSR II (BD Biosciences, San Jose, CA) flow cytometer, and the data were analyzed with FlowJo analysis software (Tree Star Inc., Ashland, OR). Side scatter and forward scatter parameters and fixable live-dead Zombie-Aqua dye were used to define live cell gates. All antibodies were purchased from BD Biosciences, eBioscience (San Diego, CA), or BioLegend (San Diego, CA) unless otherwise stated. Cells were stained with Zombie-Aqua dye (1 : 200) for 20 minutes and room temperature, washed once with FACS buffer (1x PBS, 0.3% BSA), and then cell surface blocked with Fc block (anti-CD16/anti-CD32) for 20 minutes at 4°C, prior to staining with fluorochrome-conjugated antibodies. For detecting ILC2 cells, cells were stained with Zombie-Aqua dye and Fc block as described above prior to staining with the following surface markers: FITC-conjugated lineage markers (FITC-conjugated anti-CD3, anti-CD19, anti-Ter119, anti-CD11b, and anti-F4/80), PE-conjugated T1/ST2, PE-Cy5-conjugated anti-Sca-1, PE-Cy7-conjugated anti-CD25, AlexaFlour700-conjugated anti-CD90.2, and APC-Cy7-conjugated anti-CD45. Cells were surface stained for 30 minutes at 4°C. For IL-5 and IL-13 intracellular staining, cells were stained with PE-AlexaFluor610-conjugated anti-IL-13 and PE-conjugated IL-5 (BioLegend). Cells were surface stained for 30 minutes at 4°C, prior to intracellular staining for 30 minutes at 4°C in 1x Perm/Wash buffer (BD Biosciences), with washes in 1x Perm/Wash between intracellular staining steps. Cells were then washed with 1x PBS/0.3% BSA prior to analysis on an LSR II flow cytometer. For isolation of mouse lung ILC2 cells, lineage-negative (CD3^−^, CD19^−^, Ter119^−^, CD11b^−^, and F4/80^−^) T1/ST2^+^ CD25^+^ cells were flow sorted using a BD Aria III (BD Biosciences).

### 2.7. IL-33 siRNA Knockdown and Wild-Type/IL-33-/- Murine Lung Fibroblast Generation

For *in vitro* IL-33 knockdown, 1 × 10^6^ C10 epithelial cells or murine fibroblast cells (MLF cells) were transfected with 5 *μ*M SMARTpool ON-TARGETplus IL-33 siRNA pool or scrambled siRNA control pool in 6-well costar plates using DharmaFECT transfection reagents as per the manufacturer's instructions (Dharmacon Inc., Chicago, IL). After 24 hours, cells were stimulated with 10 ng/ml of murine Oncostatin M (OSM) or remained untreated (control) for an additional 24 hours followed by collection of RNA using a PureLink RNA Mini Kit (Thermo Fisher Scientific). Transfections were done in triplicate, and extracted RNA was examined by qPCR or NanoString Technologies as described above.

Wild-type and IL-33-/- C57Bl/6 or BALB/c mouse lung fibroblast cultures were derived from mouse whole lungs by culturing minced whole lung tissue (minced using surgical scissors) in DMEM-F15 media containing 10% fetal bovine serum in wells of 6-well costar culture plates. Minced lung tissue was cultured for 1 week and nonadherent cells removed. Adherent cells were trypsinized and subcultured and 1 × 10^6^ cells stimulated for 24 hours with 10 ng/ml mouse OSM or remained untreated (control), prior to RNA extraction (using a PureLink RNA Mini Kit) and analysis of IL-33 expression by qPCR and an array of 19 genes (listed in Supplemental Figures S3 and S5) by NanoString Technologies. Nanostring counts were normalized to the expression of three housekeeping genes: beta-actin, PPia, and Ywhaz.

### 2.8. Statistics

Data were analyzed using GraphPad Prism version 8 software and presented as mean + /−standard error of the mean (SEM). For *in vivo* experiments, five to seven animals per group were utilized. One-way analysis of variance (ANOVA) with Tukey's multiple comparison test was used to determine statistical significance, which was defined as *p* < .05 using GraphPad Prism. The *p* values are indicated in the individual figures.

## 3. Results

**Figure 1:**
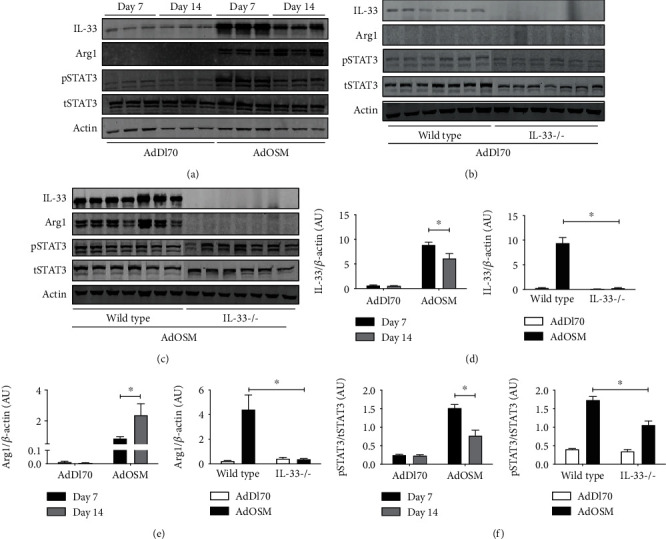
AdOSM-induced upregulation of arginase-1 (Arg1) protein expression in the mouse lung is IL-33-dependent. C57Bl/6 mice were endotracheally administered AdDl70 (control) or AdOSM and whole lung cell lysates analyzed for the expression of IL-33, phospho-STAT3/total STAT3 (pSTAT3/tSTAT3), and arginase-1 (Arg1) after 7 and 14 days by Western blotting. (a) IL-33, Arg1, phospho-STAT3 (pSTAT3), and beta-actin (Actin) expression in C57Bl/6 wild-type mice. (b, c) IL-33, pSTAT3/tSTAT3, Arg1, and beta-actin (Actin) expression in C57Bl/6 wild-type or IL-33-/- (IL-33-/-) mice at day 7. (d–f) Densitometry analysis of IL-33 and Arg1 signal relative to beta-actin levels or pSTAT3 relative to total STAT3 (tSTAT3), in C57Bl/6 mouse lung data shown in (a) or (b/c), respectively. AU: arbitrary units; *N*: 3 mice/group in (a) and 5-7 mice/group in (b/c). ^∗^*p* < .01.

**Figure 2:**
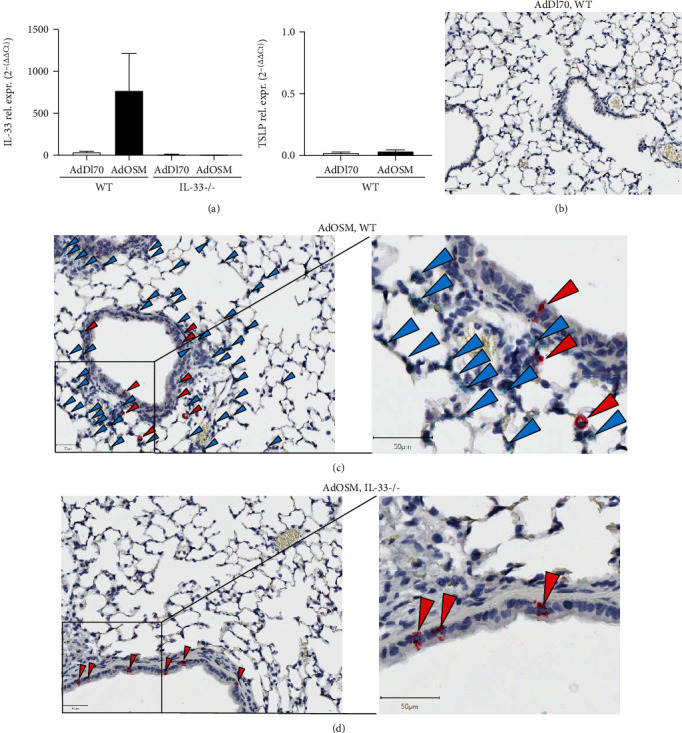
AdOSM-induced upregulation of mouse lung IL-33 mRNA and localization of IL-33 and OSM-mRNA expressing lung cells. C57Bl/6 wild-type (WT) or IL-33-/- mice were endotracheally administered AdDl70 (control) or AdOSM and analyzed after 7 days for IL-33 and TSLP mRNA expression from whole lung by qPCR (a). IL-33 and OSM mRNA expression was assessed within histological lung sections by chromatographic in situ hybridization (CISH) in AdDl70 (b), AdOSM in WT (c, right panel showing higher magnification) or AdOSM in IL-33-/- mice (d, right panel showing higher magnification). Red arrowheads: OSM+ mRNA signal; green arrowheads: IL-33+ mRNA signals.

We have previously shown that pulmonary overexpression of OSM upregulates expression of IL-33 in the lungs of C57Bl/6 and BALB/c mice in alveolar epithelial cells *in vivo* and in mouse lung epithelial cell lines *in vitro* [14]. In Figure 1, we examined the expression of IL-33, phospho-STAT3, and arginase-1 protein from whole lung extracts of mice endotracheally administered AdOSM or AdDl70 (control virus) and culled after 7 or 14 days in wild-type and IL-33-/- mice. As shown by Western blot and densitometry analysis, AdOSM induced a robust increase in the (normalized) fold change of IL-33 protein expression in the lung (approx. 14 folds at Day 7, approx. 10 folds at Day 14,), an increase in phospho-STAT3 at Day 7, an increase in arginase-1 (Arg1, a marker of M2-like macrophages) at Day 7, and further at Day 14 as compared to the AdDl70 control vector. IL-33 deficiency markedly abrogated Arg1 protein induction with a modest effect on phospho-STAT3 levels (Figures 1(b) and 1(c); densitometry shown in Figures 1(d)–1(f), right panels). IL-33 protein was absent in all KO mouse samples confirming the phenotype. The full-length form of IL-33 has been shown to be bioactive, and IL-33 is known to be cleaved into “mature” forms with varied bioactivity [26, 27]. In our system here with OSM overexpression, we observed upregulation of the levels of full-length IL-33 (detected at approximately 35 kD by Western blots) in the mouse whole lung and in the mouse C10 epithelial cell line (Supplementary Figure S1). Induction of IL-33 mRNA (but not TSLP)at Day 7 in total lung homogenates and in tissues *in situ* is shown in Figure 2. AdOSM induced robust increases in total IL-33 mRNA in wild-type mice (Figure 2(a)) while levels were undetectable in IL-33-/- mice. In sections stained for mRNA signal by CISH (representative micrographs shown), neither OSM nor IL-33 signals were detected in AdDl70-treated mice (Figure 2(b)). AdOSM mRNA signals (red) were present in epithelial and mononuclear cells in AdOSM-treated wild-type (Figure 2(c)) or IL-33-/- mice (Figure 2(d)), as expected since adenovirus vectors infect epithelial cells. IL-33 mRNA signals (green) were evident in many cells in the parenchyma or wild-type mice but were absent in IL-33-/- mice. The cells positive for IL-33mRNA are consistent with a previous work showing that type II alveolar epithelial cells stain strongly for IL-33 protein by IHC in AdOSM-treated C57Bl/6 mice [13].

**Figure 3:**
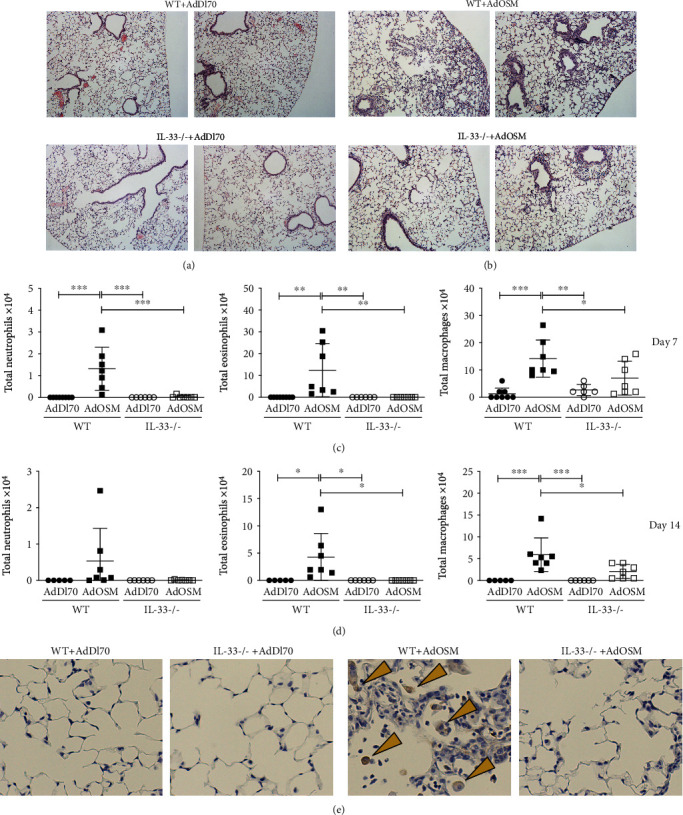
IL-33 is required for AdOSM-mediated lung inflammation. C57Bl/6 mice were endotracheally administered AdDl70 (control) or AdOSM and analyzed after 7 or 14 days. Representative H&E lung sections are shown from two different mice. (a) Wild-type (WT; top) or IL-33-/- (bottom) mice infected with AdDl70. (b) Wild-type (WT; top) or IL-33-/- (bottom) mice infected with AdOSM. (c) Total cells collected from bronchoalveolar lavage (BAL) fluid from C57Bl/6 mice at Day 7 (c) or Day 14 (d). Hema-3 staining of cytospin slides of BAL cells was done to distinguish neutrophils, eosinophil, and macrophage cell populations. ^∗^*p* < .5, ^∗∗^*p* < .01, ^∗∗∗^*p* < .001. *N* = 5‐7/group. (e) Representative images of arginase-1 (Arg1) immunohistochemistry staining (brown arrows) of lung sections from C57Bl/6 wild-type and IL-33-/- mice treated as described in (a).

In examining histopathology (shown in Figure 3(a)), wild-type and IL-33-/- C57Bl/6 mice showed normal lung architecture after AdDl70 treatment. AdOSM-treated wild-type mice showed disruption of the lung architecture, thicker alveolar walls, and increased cellular accumulation compared to AdDl70-treated counterparts (Figure 3(b)). In contrast, IL-33-/- mice administered AdOSM showed fewer signs of lung injury or altered lung architecture, indicating that AdOSM-induced histopathology is in part IL-33-dependent. Cell content of bronchoalveolar fluid (BALF) showed significant increases in neutrophil, macrophage, and eosinophil numbers in response to AdOSM that was markedly reduced in IL-33KO animals (Figure 3(c)). We did not observe any significant increases in lymphocytes in the BALF. The reduction of inflammatory cell content of BALF in AdOSM-treated IL-33-/- mice was also evident at Day 14 (Figure 3(d)). Mice-administered AdOSM also showed increased numbers of Arg1+ macrophages that were not detected in the IL-33-/- strain (Figure 3(e)).

**Figure 4:**
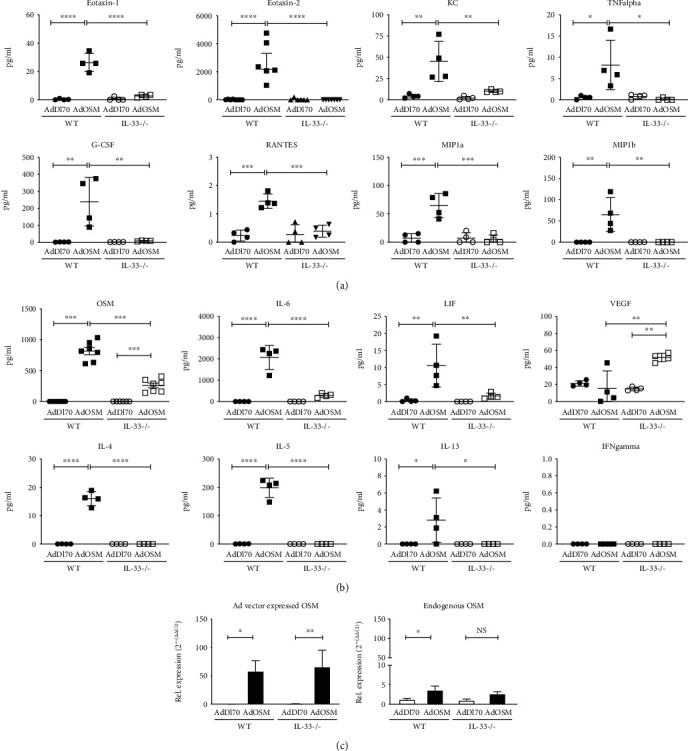
Inflammatory cytokine and Th2 cytokine induction by AdOSM is markedly attenuated in IL-33-/- (KO) mice. C57Bl/6 wild-type (WT) or IL-33 knockout mice were endotracheally administered AdDl70 (control) or AdOSM, sacrificed at day 7, and collected bronchoalveolar lavage fluid analyzed by ELISA for inflammatory cytokines/chemokines, Th1/Th2 cytokines, IL-6, and VEGF. (a) Detection of chemokines for eosinophils (eotaxin-1 and eotaxin-2) and neutrophils (KC) as well as other cytokines including TNFalpha, G-CSF, RANTES, or macrophage (MIP1a/MIP1b) in BALF of C57Bl/6 wild-type and IL-33-/- mice. (b) Detection of OSM, interleukin- (IL-) 6, leukemia inhibitory factor (LIF), VEGF, or Th2 (IL-4, IL-5, and IL-13) or Th1 (IFN*γ*) cytokines in BALF of C57Bl/6 wild-type and IL-33-/- mice. (c) Endogenous (left panel) or adenoviral-derived (right panel) expression of OSM mRNA was examined by quantitative real-time PCR analysis of whole lung tissue from C57Bl/6 wild-type and IL-33-/- mice. Closed circles: WT/AdDl70; closed squares: WT/AdOSM; open circles: IL-33KO/AdDl70; open squares: IL-33KO/AdOSM; Rel. expression: relative expression. Represents one of two separate experiments. ^∗^*p* < .05, ^∗∗^*p* < .01, ^∗∗∗^*p* < .001, and ^∗∗∗∗^*p* < .0001. *N* = 4/group.

Cytokine/chemokine levels in BALF upon AdOSM-induced lung inflammation in wild-type and IL-33-/- C57Bl/6 mice are shown in Figure 4. At Day 7 after AdOSM treatment, levels of eotaxin-1 and eotaxin-2 (eosinophil chemokine), KC (neutrophil chemokine), and MIP1alpha and MIP1beta (macrophage chemokines), were upregulated by AdOSM in wild-type mice and were markedly reduced or undetectable in IL-33-/- animals (Figure 4(a)). AdOSM-induced levels of inflammatory cytokines TNFalpha, G-CSF, RANTES, MIP1a, and MIP1b (Figure 4(a)) and the gp130 cytokines IL-6 and LIF were also significantly reduced in IL-33-/- mice (Figure 4(b)). In contrast, expression of VEGF remained unchanged due to AdOSM compared to AdDl70 (control) treatment but did show an increase in AdOSM-treated IL-33-/- mice (Figure 4(b)). Levels of typical Th2 and Th1 (IFN*γ*) cytokines were also assessed (Figure 4(b), lower panels), where AdOSM stimulated increases in IL-4, IL-5, and IL-13 in C57Bl/6 wild-type mice that was not detectable in IL-33-/- mice. IFN*γ* was not detected in BALF from either wild-type or IL-33-/- mice.

Since IL-33 deficiency appeared to have a global effect on the ability of AdOSM to induce lung inflammation, we assessed the effect of IL-33 deficiency on OSM protein levels and OSM mRNA (Ad virus-encoded and endogenous). As shown in Figure 4(c), the level of OSM protein in BALF was markedly reduced in C57Bl/6 IL-33-/- mice compared to the wild type. Using DNA primers and TaqMan hybridization probe sequences designed to be specific for either endogenous or adenoviral-derived OSM, qRT-PCR showed high levels of virus-encoded OSM mRNA in AdOSM-treated wild-type mice that was not altered by IL-33-/- deficient mice (Figure 4(c)). Endogenous OSM mRNA levels were low and showed a modest increase compared to AdDl70-infected animals.

**Figure 5:**
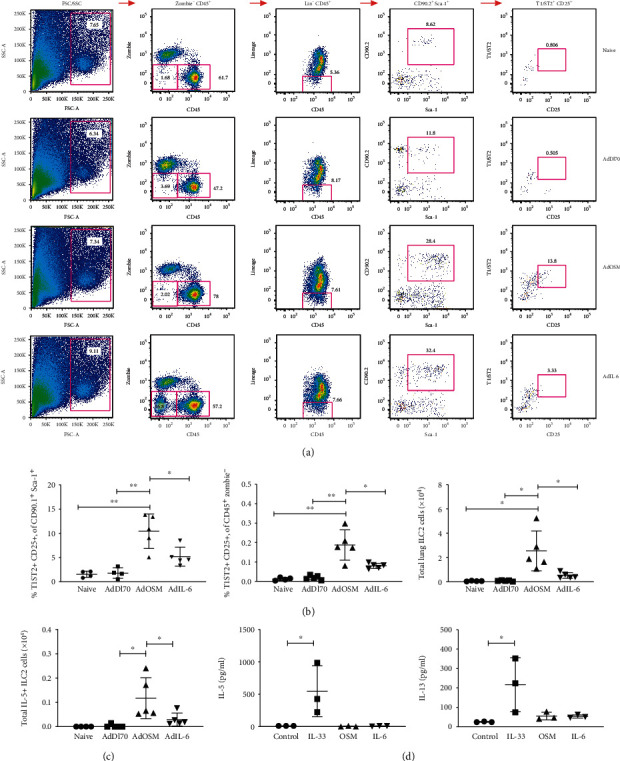
AdOSM, but not AdIL-6, stimulates accumulation of CD25+ T1/ST2 ILC2 cells. C57Bl6 mice were endotracheally administered AdDl70 (control), AdOSM, or AdIL-6, sacrificed at day 7, and ILC2 cell accumulation examined by flow cytometry of whole lung cells. ILC2 cells were assessed by flow cytometry as being lineage-negative (CD3-, CD19-, NK1.1-, Ter119-, CD11b-, and F4/80-), CD45+, CD90.2+, Sca-1+, and T1/ST2+ (IL-33R) cells that expressed varying levels of CD25. (a) Representative flow cytometry plots showing gating strategy for detecting T1/ST2+ CD25+ ILC2 cells from naive, AdDl70, AdOSM, or AdIL-6 infected mice. (b) Frequency of T1/ST2+ CD25+ CD90+ Sca-1+ parent and T1/ST2+ CD25+ CD45+ Zombie- cells, and total lung cell numbers of T1/ST2+ CD25+ ILC2 cells in the mouse lungs of naive and adenoinfected animals. (c) Total IL-5+ T1/ST2 CD25+ ILC2 cells from mouse whole lung. (d) Flow cytometry-sorted ILC2 cells from naive mouse lungs were stimulated in vitro with IL-2 alone (control) or in combination with IL-33, OSM, or IL-6 and assessed for IL-5 or IL-13 from supernatants by ELISA. One-way ANOVA with Tukey's multiple test. ^∗^*p* < .01, ^∗∗^*p* < .001. *N* = 3‐5/group.

IL-33 stimulates innate lymphoid type-2 cells (ILC2 cells) to upregulate the expression of the Th2 cytokines IL-5 and IL-13 [28, 29]. We assessed whether AdOSM (and/or AdIL-6 as a comparator) could increase the frequency of ILC2 cells in the mouse lung that could contribute to Th2 cytokine expression that we detect in this C57Bl/6 model. Flow cytometry was used to assess ILC2 cells in whole lung single cell suspensions where ILC2 cells were defined as lineage-negative (CD3-, CD19-, NK1.1-, Ter119-, CD11b-, and F4/80-) CD45+, CD90.2+, Sca-1+, T1/ST2+ (IL-33R) cells (gating strategy shown in Supplementary Figure S2). As the expression of CD25 can vary on these cells, we also assessed CD25 expression. As shown in Figure 5(a), mice treated with AdOSM had an increased frequency of T1/ST2+ CD25+ ILC2 cells as compared to AdDl70-treated or naïve mice, while AdIL-6 showed small changes in lung ILC2 cells that were not statistically significant (Figure 5(b)). AdOSM-treated animals had increased numbers of IL-5+ ILC2 cells, as compared to AdDl70- or AdIL-6 treated mice (Figure 5(c)), and small increases in IL-13+ ILC2 cells were observed but were not statistically significant (data not shown). To assess whether OSM could directly stimulate ILC2 cells, we flow cell-sorted ILC2 cells from wild-type naïve mice and treated them *ex vivo* with recombinant mouse OSM, as well as IL-6 or IL-33 as comparators. Only IL-33 was capable of inducing IL-5 or IL-13 secretion (Figure 5(d)) suggesting that OSM or IL-6 alone cannot directly regulate ILC2 cells.

**Figure 6:**
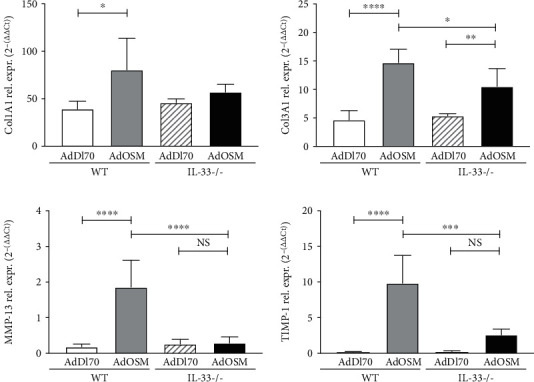
Col1A1, Col3A1, matrix metalloproteinase-13 (MMP-13) and tissue inhibitor of matrix metalloproteinase-1 (TIMP-1) expression in the lungs of wild-type and IL-33-/- mice. C57Bl/6 wild-type (WT) or IL-33-/- mice were endotracheally administered AdDl70 (control) or AdOSM, sacrificed at day 7, and lung homogenates examined for mRNA expression of extracellular matrix genes Col1A1, Col3A1, and matrix proteinase and proteinase inhibitor genes MMP-13 and TIMP-1. Expression was assessed by quantitative real-time PCR. Rel. expr.: relative expression. ^∗^*p* < .05, ^∗∗^*p* < .01, ^∗∗∗^*p* < .001, ^∗∗∗∗^*p* < .0001. *N* = 5/group.

We then tested whether IL-33 was required for lung matrix gene expression and MMP and TIMP-1 genes [22, 30]. As shown in Figure 6, AdOSM upregulated the expression of lung matrix genes col1A1 and col3A1 and MMP-13 and TIMP-1 in wild-type C57Bl/6 whole lung homogenates. While the AdOSM induction of col1A1 showed a trend of reduction, col3A1, MMP-13, and TIMP-1 mRNA levels were significantly reduced in C57Bl/6 IL-33-/- mice.

**Figure 7:**
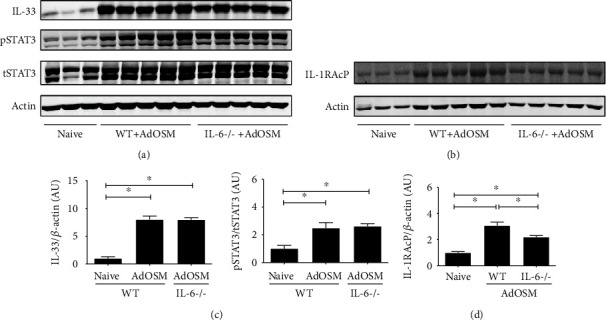
AdOSM-induced IL-33 expression is independent of IL-6. C57Bl/6 wild-type or IL6-/- mice were endotracheally administered AdOSM and whole lung cell lysates analyzed the expression of beta actin and (a) IL-33, phospho-STAT3/total STAT3 (pSTAT3/tSTAT3), or (b) IL-1 receptor accessory protein (IL-1RAcP), after 7 days by Western blotting. (c, d) Densitometry analysis of IL-33 and or IL-1RAcP (relative to actin) and of pSTAT3/tSTAT3 (c) (d). AU = arbitrary units. N =5/group. ^∗^*p* < .01.

We here found that AdOSM-induced Arg1 protein (Figure 1) and Arg1+ cell accumulation (Figure 3(e)) requires IL-33 and previously found a requirement of IL-6 in the same system by using IL-6KO mice [21]. We thus assessed whether the upregulation of IL-33 by AdOSM was also dependent on IL-6. Whole lung cell homogenates prepared from wild-type or IL-6-deficient mice treated with AdOSM for 7 days (Figure 7) showed elevated levels of IL-33, phospho-STAT3, and the IL-33 receptor subunit IL-1RAcP in wild-type mice by AdOSM. IL-6 deficiency did not affect the elevated levels of IL-33 or pSTAT3 (Figures 7(a) and 7(b)), or ST-2 (data not shown), but showed a modest decrease in IL-1RAcP protein levels. Quantitative densitometry analysis is shown in in Figures 7(b) and 7(d). Thus, AdOSM-mediated IL-33 expression appears to be independent of IL-6.

**Figure 8:**
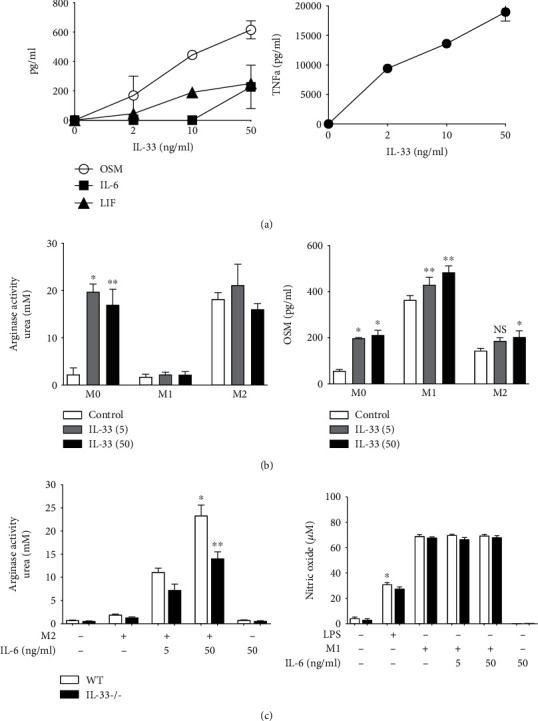
IL-33 stimulates production of IL-6-type cytokines (OSM, IL-6, and LIF) and arginase-1 activity from macrophages. (a) RAW264.7 cells were stimulated with varying doses of recombinant mouse IL-33 (0-50 ng/ml). After 48 hours, cell culture media was collected and assayed for the presence of OSM, IL-6, LIF, and TNFalpha (TNFa) by ELISA. Four replicates/treatment. (b) Bone marrow-derived macrophages (BMDM) were differentiated into M1- (LPS+IFN*γ*) or M2-type (IL-4+IL-13) macrophages or remained untreated (M0) and stimulated with 5 or 50 ng/ml of IL-33 for 24 hours. Cell lysates were assayed for arginase-1 activity (urea production) and supernatants assayed by ELISA for Oncostatin M (OSM). (c) BMDM cells from wild-type or IL-33-/- mice were differentiated into M1 or M2 cells or treated with LPS alone with or without IL-6 (a potentiator of M2 cells) and assayed for arginase-1 activity or nitric oxide production. Four replicates/treatment. ^∗^*p* < .01, ^∗∗^*p* < .001.

In addition to Th2 T cells and ILC2 cells, macrophage populations may be responsive to IL-33 and contribute to OSM-induced lung inflammation. We thus assessed the RAW264.7 macrophage cell line (which expresses both IL-33 receptor subunits [31]) and bone marrow-derived macrophages (BMDM). As shown in Figure 8(a), IL-33 stimulated significantly increased levels of OSM (up to 600 pg/ml) as well as LIF and IL-6 to a lesser degree (up to 200 pg/ml of LIF or IL-6) in a dose-dependent manner, as well as in TNFalpha in parallel, in RAW264.7 cells. BMDM cells cultured in conditions for M1-skewing (LPS/IFN*γ*), M2-skewing (IL-13/IL-4) or unskewed cells (M0 cells) were stimulated with IL-33 (5 and 50 ng/ml). In unskewed (M0) BMDM cells, IL-33 induced a statistically significant increase in arginase-1 activity and production of OSM (Figure 8(b)). M2-differentiated BMDM cells also showed elevated levels of arginase-1 activity (as compared to untreated BMDM cells) that was not further increased by IL-33 treatment. OSM was induced in either M1 or M2 skewing conditions, and this was augmented by IL-33 (Figure 8(b), right panel). IL-33 deficiency also compromised maximal arginase-1 activity that was achieved when M2 BMDM cells were cultured in the presence of IL-6 (Figure 8(c)) whereas NO induction was not affected. Thus, IL-33 is capable of regulating expression of OSM and other gp130 cytokines.

Since IL-33 is localized to the nucleus [32] and there has been a suggestion of nuclear roles of IL-33 as a transcriptional repressor [33, 34], we examined mouse lung fibroblast (MLF) cultures derived from wild-type and IL-33KO mice. We found no differences between wild-type and IL-33KO cell cultures in their transcriptional response to OSM in examining 19 OSM-responsive mRNAs (Supplemental Figures S3). Similar results were observed in IL-33 siRNA-knockdown cultures of lung fibroblasts, derived from C57Bl/6 mice or C10 mouse epithelial cells (Supplemental Figures S4 and S5).

**Figure 9:**
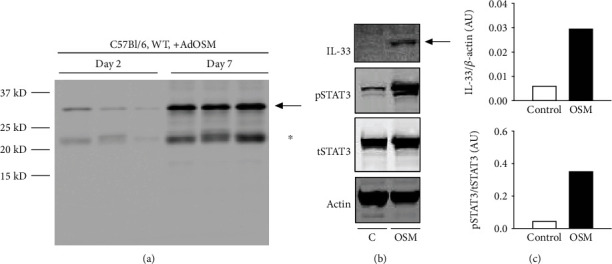
Comparison of AdOSM-induced IL-33 expression in C57Bl/6 mouse lung using the Nessy-1 monoclonal antibody and OSM-induced IL-33 upregulation in Human Type 2 Alveolar Epithelial Cells (A549). (a) C57Bl/6 wild-type (WT) or IL-33 knockout (IL-33KO) mice were endotracheally administered AdDl70 (control) or AdOSM for 7 days, and whole lung cell lysate was analyzed for the expression of IL-33 by Western blotting using the Nessy-1 monoclonal antibody. (b) 300,000 A549 cells/well in 6-well plates were stimulated with human OSM (20 ng/ml) for 24 hours, and Western blot analysis was performed for detection of IL-33 (Nessy-1), pSTAT3 (NEB Cell Signaling), tSTAT3 (NEB Signaling), and actin (Santa Cruz) from cell lysates. Black arrow indicates migration of IL-33-specific band. ^∗^Mouse IgG light chain. (c) Densitometry of IL-33 and pSTAT3/tSTAT3 signal was completed using Image Studio Lite for samples in (b). AU: arbitrary units.

Since we did not detect mature IL-33 at Day 7, we assessed lung extracts at Day 2 to determine if mature IL-33 was evident earlier in the time course of AdOSM treatment. As shown in Figure 9(a), Day 2 whole lung cell homogenates from C57Bl/6 mice that were endotracheally administered AdOSM showed lower levels of IL-33 proform protein as compared to Day 7, and there was no species of smaller molecular weight corresponding to mature IL-33 detected by Western blots. To determine if OSM induces proforms and/or mature forms of IL-33 in human systems, we used A549 human alveolar type II epithelial cell cultures stimulated in vitro (Figures 9(b) and 9(c)) and observed that human OSM induced a 35 kD signal (proform size). We did not detect lower molecular weight species by Western blots in A549 cells (Supplementary Figure S6).

## 4. Discussion

We show here that IL-33 is required for induction of a Th2-like inflammatory response in the lungs of C57Bl/6 mice endotracheally administered AdOSM. In IL-33-deficient mice, the AdOSM-induced elevation of Th2 cytokines (IL-4, IL-5, and IL-13) was absent, inflammatory cytokines (IL-6, MIP1a/b, TNFalpha, and KC) were markedly diminished, and AdOSM-induced eosinophil, neutrophil, and AA/M2 (as measured by Arg1+ cells) accumulation was similarly reduced. AdOSM-induced expression of extracellular matrix proteins and their inhibitors was also reduced by IL-33 deficiency in C57Bl/6 mice. AdOSM could induce levels of Th2-cytokine-producing ILC2 cells and induces IL-33 by an IL-6-independent pathway. We also show here that IL-33 can regulate expression of OSM, and other gp130 cytokines IL-6 and LIF, in macrophages. Similar to the mouse system, where OSM can directly stimulate IL-33 in type II alveolar epithelial cells [13], human OSM can also induce expression of IL-33 in A549 lung epithelial cells in vitro (Figure 9). Full-length IL-33 is bioactive and can be cleaved (through apoptosis) to generate inactive forms [35]. In our study here, we detect only full-length IL-33 induced by OSM by Western blots as the major product, suggesting this form is primarily responsible for activity in this system.

Previous work has shown OSM to acutely induce a Th2-like environment in the lungs of C57Bl/6 mice [36], and OSM has been detected in a variety of Th2-mediated mucosal abnormalities such as asthma and allergy [37, 38]. Our findings here that OSM stimulates IL-33 expression and that IL-33 is required for OSM induction of Th2-like factors are consistent with the previously established role of IL-33 as a mediator of Th2-like responses [7, 39]. Our results suggest that an OSM-IL-33 axis and subsequent IL-33-mediated sequelae may participate in innate immune mechanisms of generation of Th2/M2-like inflammation without the requirement of antigen-specific adaptive Th2 cells. A subset of asthma patients are nonatopic but do show a Th2-skewed inflammatory phenotype [40], which may be driven by such an OSM-IL-33 mechanism.

ILC2 cells have also been shown to be important mediators for driving Th2 responses [29, 41]. T1/ST2, an IL-33 receptor component, is a primary identifying marker of ILC2 cells, and stimulation of ILC2 cells with IL-33 results in the production of Th2 cytokines (IL-5 and IL-13) by these cells [42–46]. ILC2 cells also intrinsically express basal levels of IL-5 and the M2 macrophage marker arginase-1 [47, 48]. Here, we observed that AdOSM could increase the number of ILC2 cells in the (C57Bl/6) lung that appears to be indirect via the induction of IL-33 by OSM, since recombinant IL-33, and not recombinant OSM or IL-6, stimulated production of Th2 cytokines *in vitro* by ILC2 cells isolated from the naïve mouse lung (Figure 5(d)). As the absence of IL-33 limits egress of ILC2 cells from the bone marrow [49], we were not able to directly test whether AdOSM induces an increase in ILC2 cells in IL-33-/- mice in vivo. The use of neutralizing antibodies to deplete (Th2) T cells or ILC2 cells may be alternative approaches for assessing the relative contribution T cells verses ILC2 cells to the AdOSM-induced Th2-like effects observed. It is worth noting that Rag-/- mice still contain ILC2 cells [45]. Thus, although ILC2 cell numbers are increased by AdOSM *in vivo* (Figure 5), that Rag-/- mice lack an OSM-induced Th2-like phenotype [18] suggests that Th2 T cells may contribute to AdOSM-induced Th2 cytokine responses, although it is not clear if ILC2 cells in Rag-/- are fully functional. Alternatively, there may be other cell sources of IL-4, IL-5, and IL-13 that are induced by OSM in the absence of antigen specificity that are not yet identified.

Arginase-1 is a common identifying marker of M2 alternatively activated macrophages [50]. These M2 macrophages are activated by Th2 cytokines (IL-4/IL-13) to produce arginase-1 and ECM remodelling proteinases or proteinase inhibitors [51]. This activation is further potentiated by IL-6 [21, 23]. We show here that C57Bl/6 IL-33-/- lacked AdOSM-induced expression of arginase-1 (Arg1), and AdOSM-induced increases in Arg1+ macrophages were absent in C57Bl/6 IL-33-/- as compared to wild-type mice (Figures 1 and 3(e)). This is consistent with a requirement for IL-33 to induce IL-4/IL-13 which is necessary for the differentiation of macrophages into an M2-like Arg1+ phenotype [52]. We also observed that bone marrow-derived macrophages (BMDM) induced to produce high levels of Arg1 activity, in the presence of IL-4, IL-13, and IL-6, showed a significant decrease in Arg1 activity in IL-33-/- BMDM, suggesting that in addition to IL-4/IL-13 signaling, endogenous IL-33 in BMDM may be required for maximal Arg1 induction in mouse models. Further, Dubey et al. showed that OSM did not directly induce Arg1 in mouse bone marrow-derived macrophages *in vitro* suggesting indirect effects of OSM on macrophages *in vivo* [21]. However, AdOSM-induced Arg1 did require IL-6 *in vivo* [21], which is downstream of IL-33 in the present system. Arginase-1 is one marker of AA/M2 cells but is not definitive. Recent work has shown that AdOSM induces AA/M2 markers RELM*α*+ (Fizz-1) and YM-1+ mononuclear cells in alveolar spaces but also induces expression of such markers in other cells [53]. Whether IL-33-/- affects a range of classically and/or alternatively activated macrophage populations using more definitive markers for various subsets would be the subject of future work. Arginase is also expressed by ILC2 cells [48], and AdOSM-induced increases in ILC2 could also contribute to the net arginase expression.

Although IL-6 is required for maximal Th2-like inflammation in C57Bl/6 mice [23], IL-6 was not required for AdOSM-induced IL-33 (Figure 7). This indicates that, at least in C57Bl/6 mice, AdOSM-induced IL-33 signaling is upstream of IL-6 signaling. Consistent with this, OSM can directly stimulate IL-33 in alveolar type II epithelial cells *in vitro* [13], and IL-33 induces IL-6 expression by macrophages (Figure 8(a)). We also observed that IL-33 directly stimulates the expression of OSM in RAW264.7 macrophages or in untreated BMDM cells (M0) or M1/M2-differentiated BMDM cells (Figure 8). Upon speculation, our *in vitro* data may suggest an interaction between macrophages and epithelial cells (or other IL-33-expressing cells) in that sufficient bioactive IL-33 produced by epithelial cells could feedback onto macrophages to induce OSM, which in turn further stimulates IL-33 expression in a positive feedback fashion. That IL-33 can regulate OSM and IL-6 directly in macrophage populations *in vitro* suggests that the absence of this function contributes to the lower levels of OSM and IL-6 protein in IL-33-/- mice *in vivo* (Figure 4(b)). Alternatively (or in addition), the decreased level of OSM or IL-6 protein in IL-33-/- BALF is a result of a reduced number of inflammatory macrophages and neutrophils observed in IL-33-/- mouse lungs (Figure 3(c)). Neutrophils are also a significant source of OSM [37]. Since we did not observe Ad vector-expressed OSM mRNA levels to be affected by IL-33 deficiency (Figures 4(c)), the decrease in OSM protein in BALF may reflect the lack of neutrophils in the IL-33 knockout [35, 54, 55].

Alternatively, consumption of OSM ligand may be increased in IL-33-/- mouse lungs. This does not appear to involve OSMR expression, since although AdOSM induced higher levels of OSMR*β* mRNA in mouse whole lungs, we did not observe any significant difference in the fold induction of OSMR*β* in wild-type vs. IL-33-/- mice (Supplemental Figure S7). Determination of whether OSMR protein expression may be altered awaits further analysis with validated antibodies. Another alternative explanation to why OSM levels are reduced in IL-33-/- mice could be IL-33 regulation of posttranscriptional mechanisms. IL-33 has been shown to regulate the expression of microRNAs important to the induction of inflammation and tissue repair [56, 57]. Whether IL-33 can induce expression of microRNAs that regulate OSM protein levels (and/or other IL-6-type cytokines) is not known and would require further study.

IL-33 is in large part localized to the cell nucleus and has been suggested to act as a transcriptional repressor of IL-6 in other systems [32–34]. However, we did not observe differences in IL-6 or several other OSM-inducible mRNAs in IL-33KO cells or by IL-33 siRNA knockdown (Supplemental Figures S3-S5). This is consistent with other recent publications that have also not found a role of nuclear IL-33 in regulating gene expression [24, 58]. VEGF levels were increased in the BAL in IL-33-/- which could reflect direct or indirect suppression of VEGF by IL-33 in certain cells. Both positive [59] and inversely correlated [60] effects of IL-33 on VEGF expression have been observed while OSM has been shown to upregulate expression of factors affecting VEGF expression (HIF1alpha, STAT3) [61–63]. We found that OSM induction of VEGF mRNA in MLF was not affected by IL-33 deficiency *in vitro* (as noted above, Supplementary Figures S3) suggesting again that the induced affect did not involve transcriptional regulation by IL-33 in the nucleus.

AdOSM induced the upregulation of col1A1, col3A1, MMP-13, and TIMP-1 as previously described [19, 22, 30] and was reduced in whole lung extracts of IL-33-/- mice (Figure 6). Since OSM can directly induce coll1A1, col3A1, and TIMP-1 in connective tissue cells such as lung fibroblasts *in vitro*, extracellular IL-33 may be required for the maximal direct induction of these genes by OSM. The receptor chains for IL-33 (ST-2 and IL-1Rap) are present on fibroblasts and endothelial cells, although to our knowledge, there is no evidence available at present that shows that IL-33 alone can directly regulate these genes in these cells [24, 58]. Alternatively, the reduced mRNA may reflect the reduced number of inflammatory cells in the IL-33-/- C57Bl/6 mice that contribute to the levels of mRNA of these factors in whole lung extracts. IL-33 has been proposed to participate in the development of lung fibrosis in bleomycin-induced mouse models [64], and it will be of interest to further explore whether IL-33 is involved in other models of extracellular matrix deposition in the lung [23] or different tissues.

While mouse OSM binds a distinct type-II receptor comprised of OSM receptor beta (OSMR*β*) and gp130, human OSM is known to bind either a similar high affinity type-II receptor (OSM*β*/gp130) or a lower affinity type-I receptor (comprised of the LIF receptor alpha and gp130) [15, 65]. OSM is detected in patient inflammatory diseases that are mediated by Th2 cellular mechanisms [37, 38]. The ability of human OSM to stimulate IL-33 expression in the A549 lung epithelial cell line (Figures 9(b) and 9(c)) suggests that further exploration of OSM in mouse and human systems in upregulating the expression of IL-33, a known driver of Th2-mediated pathologies [4, 66, 67], is merited.

## 5. Conclusions

Taken together, we show data to support a functional OSM-IL-33 axis that is required for AdOSM-induced Th2-like and AA/M2-like inflammation in the lungs of C57Bl/6 mice, through effects on ILC2 cell and macrophage accumulation, and ECM gene expression. This pathway may contribute to innate immune mechanisms of Th2-like disease conditions and may also contribute to exacerbation of adaptive immune responses to specific antigens that involve Th2 cytokine and M2 macrophage skewing.

## Data Availability

All original data can be made available upon request to the corresponding author Dr. Carl D. Richards through email address richards@mcmaster.ca. This includes all raw data with excel and prism files (ELISA, qRT-PCR and nanostring data), gel /immunoblot images, ISH and IHC images, and Flow cytometry files.

## Supplementary Materials

Supplementary Materials Supplemental Figure S1: immunoblot analysis detects increases in full-length pro-IL-33 in response to OSM. (A) Lung tissue homogenate protein from 3 individual BALB/c mice treated with AdDl70 (lanes 1-3) or 3 with AdOSM vector (lanes 4-6) for 7 days, or C10 alveolar epithelial cell protein lysates from unstimulated (lane 7) or OSM-stimulated cells (treated with 5 ng/ml OSM for 18 hours, lane 8) were separated by 12% SDS-PAGE. Blots were probed for IL-33 (top panel) and actin (bottom panel). Lanes 9 and 10 contained samples of 1 ng and 2 ng (respectively) recombinant mature IL-33 (R&D systems). BLUeye molecular weight marker migration is shown on the left. (B) Full length blot of IL-33 Western Blotting of lung extracts from individual mice treated as indicated. Position of IL-33 pro-form is denoted by the black arrow. Supplemental Figure S2: gating scheme and gate assignments of fluorescence-minus-one(FMO) flow cytometry staining controls used for defining Zombie^−^ (live cells), CD45^+^ lineage^−^ CD90.2^+^ Sca-1^+^ T1/ST2^+^ CD25^+^ ILC2 cells. (A) Gating scheme for defining ILC2 cells. (B) FMO cell staining and gate assignments used for defining Zombie^−^ (live cells), CD45^+^, lineage^−^, CD90.2^+^, Sca-1^+^, T1/ST2^+^, and CD25^+^ gates. Staining with or without the indicated fluorochrome conjugate is shown. Supplemental Figure S3: 1 × 10^6^ mouse lung fibroblast cell cultures derived from C57Bl/6 wild-type (WT) and IL-33-knockout (IL-33KO) mice were stimulated with murine OSM (mOSM) for either 6 or 24 hours, or remained untreated (Control). RNA was extracted using a PureLink RNA Mini Kit and OSM-responsive and non-responsive genes were accessed by Nanostring Technologies. Nanostring counts were normalized to the expression of beta-actin, PPia and Ywhaz. Supplemental Figure S4: 1 × 10^6^ C57Bl/6 murine lung fibroblast (MLF) cells (A) or C10 alveolar epithelial cells (B) were transfected with SMARTpool ON-TARGETplus IL-33 siRNA (mIL-33 siRNA) or scrambled siRNA (SCRAM) followed by stimulation with murine OSM (mOSM) for 24 hours, or remained untreated (Control). RNA was extracted using a PureLink RNA Mini Kit and mouse IL-33, TIMP-1, Col3A1, IL-6, NOS2, and IL-4 receptor alpha (IL-4Ra) accessed by quantitative PCR (relative to 18S expression). Supplemental Figure S5: 1 × 10^6^ C10 alveolar epithelial cells were transfected with SMARTpool ON-TARGETplus IL-33 siRNA (mIL-33 siRNA) or scrambled siRNA (SCRAM) followed by stimulation with murine OSM (mOSM) for 24 hours, or remained untreated (Control). RNA was extracted using a PureLink RNA Mini Kit and mouse IL-33, IL-6, leukemia inhibitory factor (LIF), TIMP-1, IL-4 receptor alpha (IL-4Ra), IL-6 receptor alpha (IL-6Ra), OSM receptor beta (OsmR) and TGFbeta1 accessed by quantitative Nanostring Technologies. Supplemental Figure S6: immunoblot detection of IL-33 in human A549 cells. Full-length blots of IL-33 Western blotting shown in Figure 9(b) using the Nessy-1 mouse monoclonal antibody (A) or when using similar whole cell lysates with the R&D goat polyclonal antibody (B). Position of IL-33 proform is denoted by the black arrow. Supplemental Figure S7: AdOSM-induced OSMR*β* expression in C57Bl6 wild-type and IL-33-/- mice. C57Bl/6 wild-type (WT) or IL-33-/- mice were endotracheally administered with AdDl70 (control) or AdOSM and analyzed after 7 days for OSM receptor beta (OSMR*β*) mRNA expression from whole lung by qPCR.
