# Supplementary material for: IL-33 Mediates Lung Inflammation by the IL-6-Type Cytokine Oncostatin M
Source: Mediators Inflamm. 2020 Nov 28;2020:4087315. doi: 10.1155/2020/4087315 (PMC7744230; doi:10.1155/2020/4087315)
Supplement: Supplementary Materials — Supplemental Figure S1: immunoblot analysis detects increases in full-length pro-IL-33 in response to OSM. (A) Lung tissue homogenate protein from 3 individual BALB/c mice treated with AdDl70 (lanes 1-3) or 3 with AdOSM vector (lanes 4-6) for 7 days, or C10 alveolar epithelial cell protein lysates from unstimulated (lane 7) or OSM-stimulated cells (treated with 5 ng/ml OSM for 18 hours, lane 8) were separated by 12% SDS-PAGE. Blots were probed for IL-33 (top panel) and actin (bottom panel). Lanes 9 and 10 contained samples of 1 ng and 2 ng (respectively) recombinant mature IL-33 (R&D systems). BLUeye molecular weight marker migration is shown on the left. (B) Full length blot of IL-33 Western Blotting of lung extracts from individual mice treated as indicated. Position of IL-33 pro-form is denoted by the black arrow. Supplemental Figure S2: gating scheme and gate assignments of fluorescence-minus-one(FMO) flow cytometry staining controls used for defining Zombie− (live cells), CD45+ lineage− CD90.2+ Sca-1+ T1/ST2+ CD25+ ILC2 cells. (A) Gating scheme for defining ILC2 cells. (B) FMO cell staining and gate assignments used for defining Zombie− (live cells), CD45+, lineage−, CD90.2+, Sca-1+, T1/ST2+, and CD25+ gates. Staining with or without the indicated fluorochrome conjugate is shown. Supplemental Figure S3: 1 × 10^6^ mouse lung fibroblast cell cultures derived from C57Bl/6 wild-type (WT) and IL-33-knockout (IL-33KO) mice were stimulated with murine OSM (mOSM) for either 6 or 24 hours, or remained untreated (Control). RNA was extracted using a PureLink RNA Mini Kit and OSM-responsive and non-responsive genes were accessed by Nanostring Technologies. Nanostring counts were normalized to the expression of beta-actin, PPia and Ywhaz. Supplemental Figure S4: 1 × 10^6^ C57Bl/6 murine lung fibroblast (MLF) cells (A) or C10 alveolar epithelial cells (B) were transfected with SMARTpool ON-TARGETplus IL-33 siRNA (mIL-33 siRNA) or scrambled siRNA (SCRAM) followed by stimulation w [file 4087315.f1.pdf]

Supplemental Figure S1

A

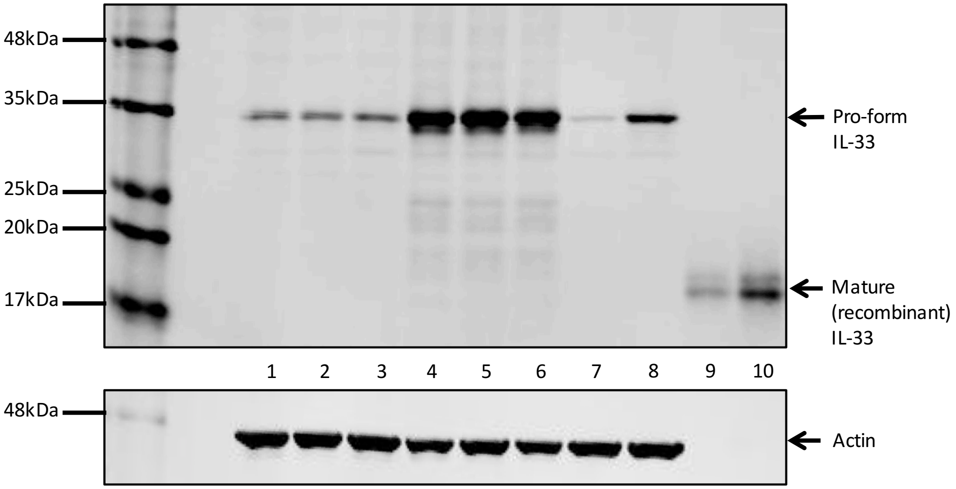

B

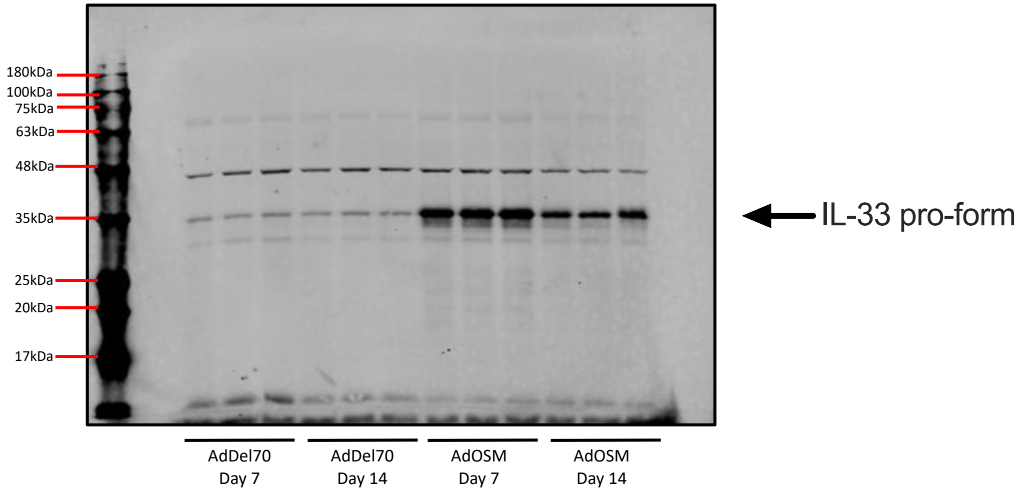

Supplemental Figure S1. Immunoblot analysis detects increases in full length pro-IL-33 in response to OSM. (A) Lung tissue homogenate protein from 3 individual BALB/c mice treated with AdDl70 (lanes 1-3) or 3 with AdOSM vector (lanes 4-6) for 7 days, or C10 alveolar epithelial cell protein lysates from unstimulated (lane 7) or OSM-stimulated cells (treated with 5 ng/ml OSM for 18 hours, lane 8) were separated by 12% SDS-PAGE. Blots were probed for IL-33 (top panel) and Actin (bottom panel). Lanes 9 and 10 contained samples of 1 ng and 2 ng (respectively) recombinant mature IL-33 (R&D systems). BLUeye molecular weight marker migration is shown on the left. (B) Full length blot of IL-33 Western Blotting of lung extracts from individual mice treated as indicated. Position of IL-33 pro-form is denoted by the black arrow.

Supplemental Figure S2

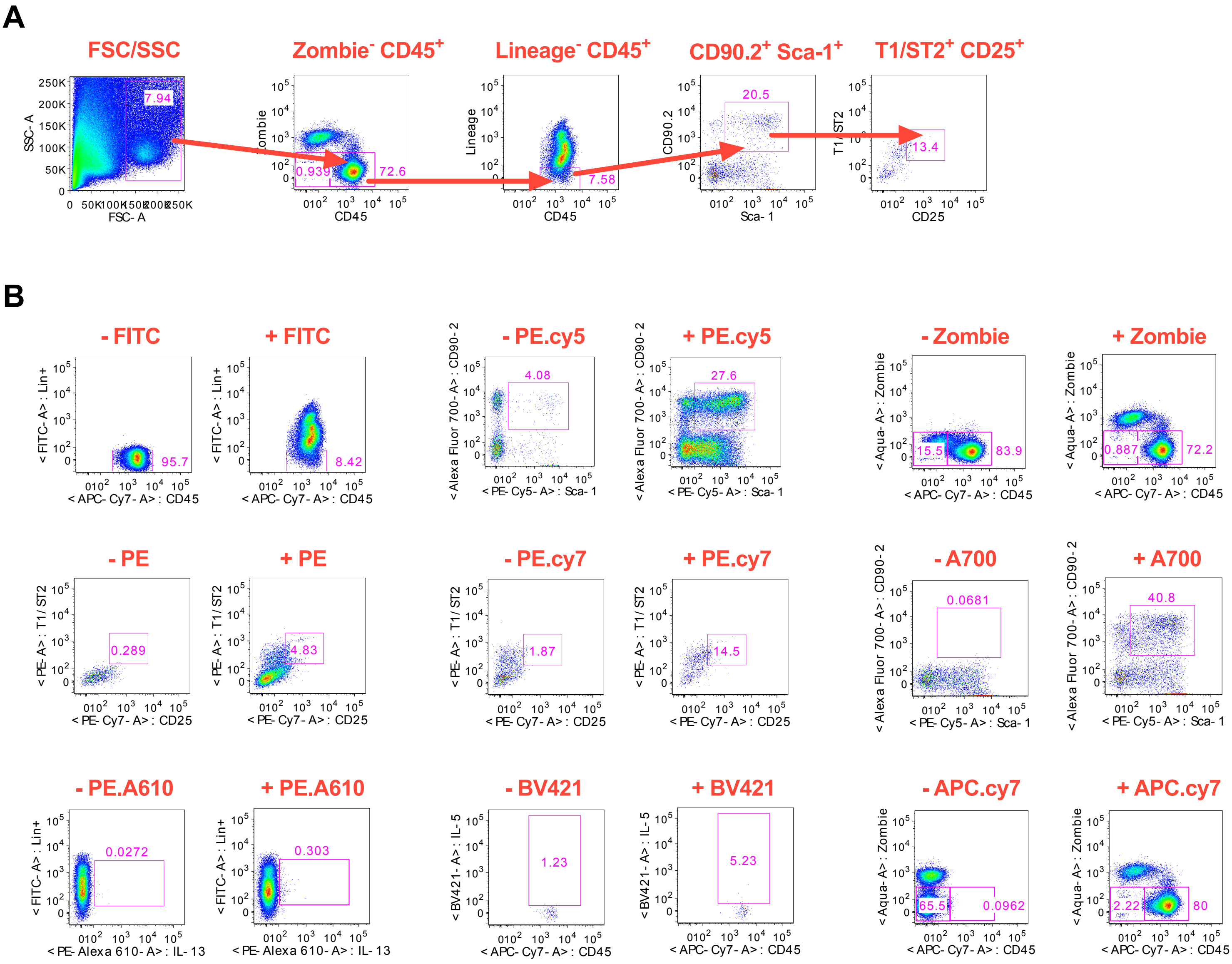

Supplemental Figure S2. Gating scheme and gate assignments of fluorescence-minus-one(FMO) flow cytometry staining controls used for defining Zombie⁻ (live cells), CD45⁺ lineage⁻ CD90.2⁺ Sca-1⁺ T1/ST2⁺ CD25⁺ ILC2 cells. (A) Gating scheme for defining ILC2 cells. (B) FMO cell staining and gate assignments used for defining Zombie⁻ (live cells), CD45⁺, lineage⁻, CD90.2⁺, Sca-1⁺, T1/ST2⁺, and CD25⁺ gates. Staining with or without the indicated fluorochrome-conjugate is shown.

Supplemental Figure S3

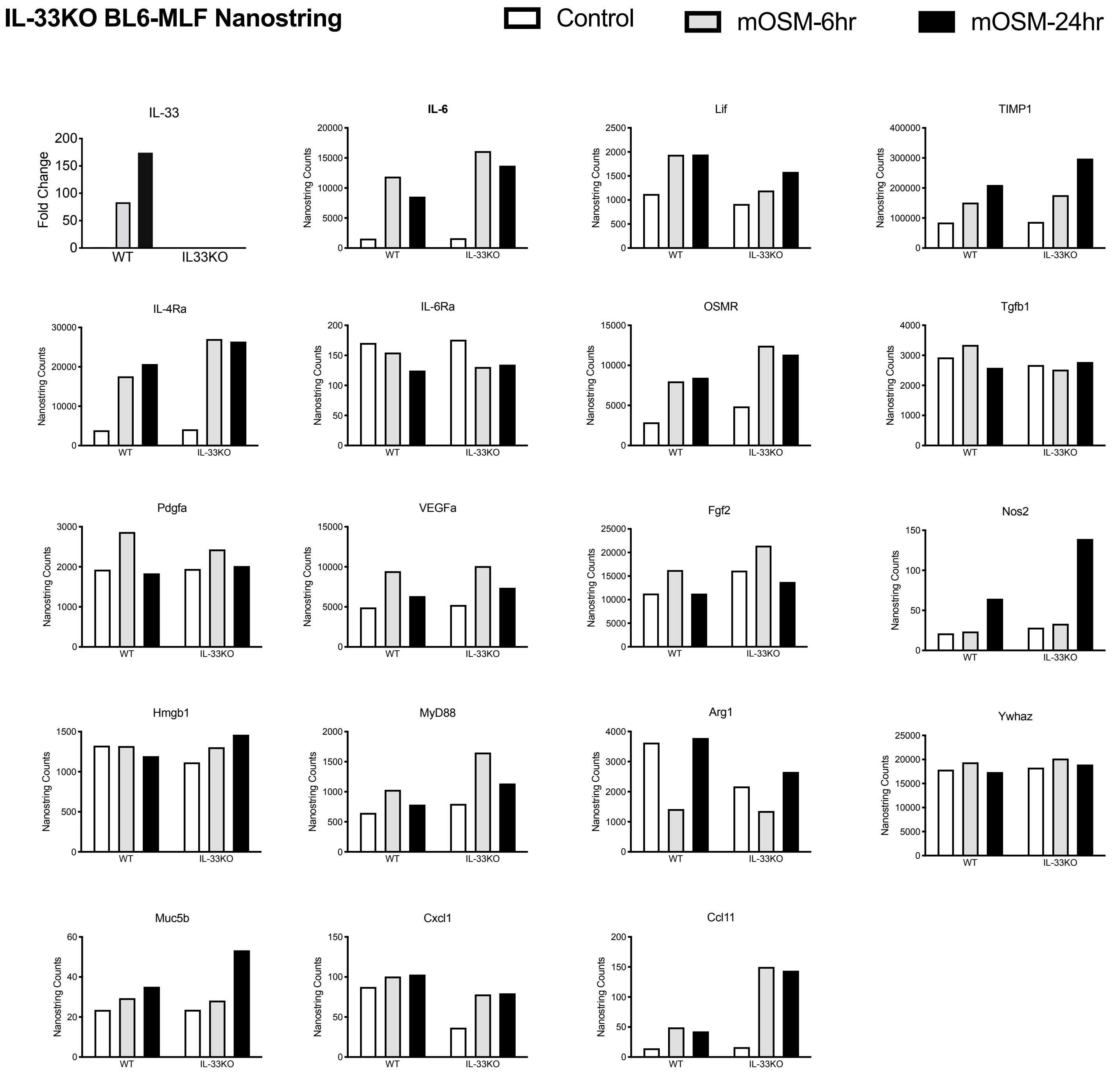

Supplemental Figure S3

1×10^6 mouse lung fibroblast cell cultures derived from C57Bl/6 wildtype (WT) and IL-33-knockout (IL33KO) mice were stimulated with murine OSM (mOSM) for either 6 or 24 hours, or remained untreated (Control). RNA was extracted using a PureLink RNA Mini Kit and OSM-responsive and non-responsive genes were accessed by Nanostring Technologies. Nanostring counts were normalized to the expression of beta-actin, PPia and Ywhaz.

Supplemental Figure S4

Control mOSM

A IL-33 siRNA + MLF cells:

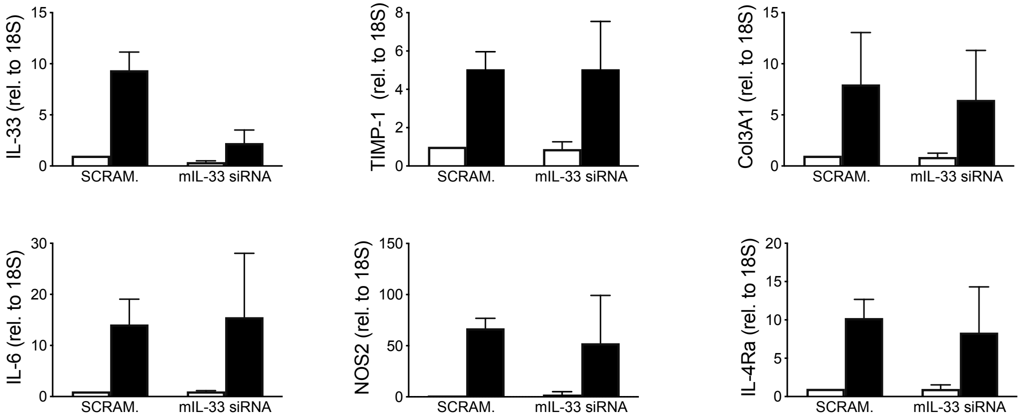

B IL-33 siRNA + C10 Alveolar Epithelial cells:

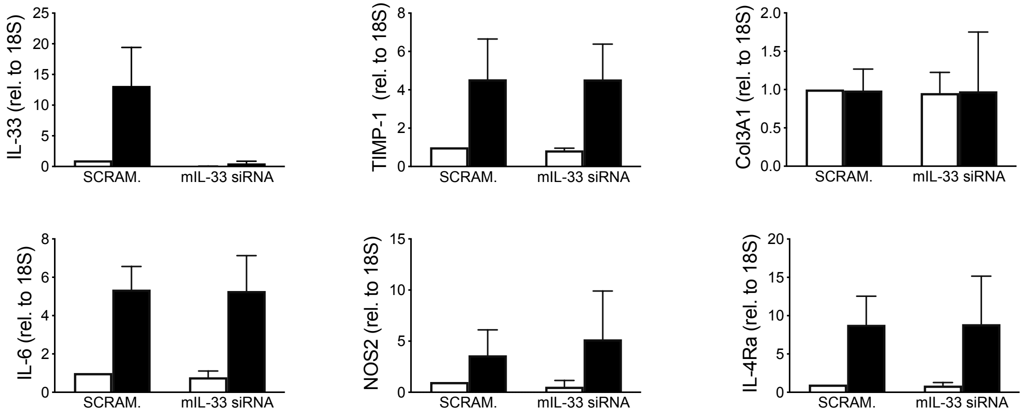

Supplemental Figure S4. $1x10^6$ C57Bl/6 murine lung fibroblast (MLF) cells (A) or C10 alveolar epithelial cells (B) were transfected with SMARTpool ON-TARGETplus IL-33 siRNA (mIL-33 siRNA) or scrambled siRNA (SCRAM.) followed by stimulation with murine OSM (mOSM) for 24 hours, or remained untreated (Control). RNA was extracted using a PureLink RNA Mini Kit and mouse IL-33, TIMP-1, Col3A1, IL-6, NOS2 and IL-4 receptor alpha (IL-4Ra) accessed by quantitative PCR (relative to 18S expression).

Supplemental Figure S5

IL-33 siRNA + C10 Alveolar Epithelial cells (Nanostring):

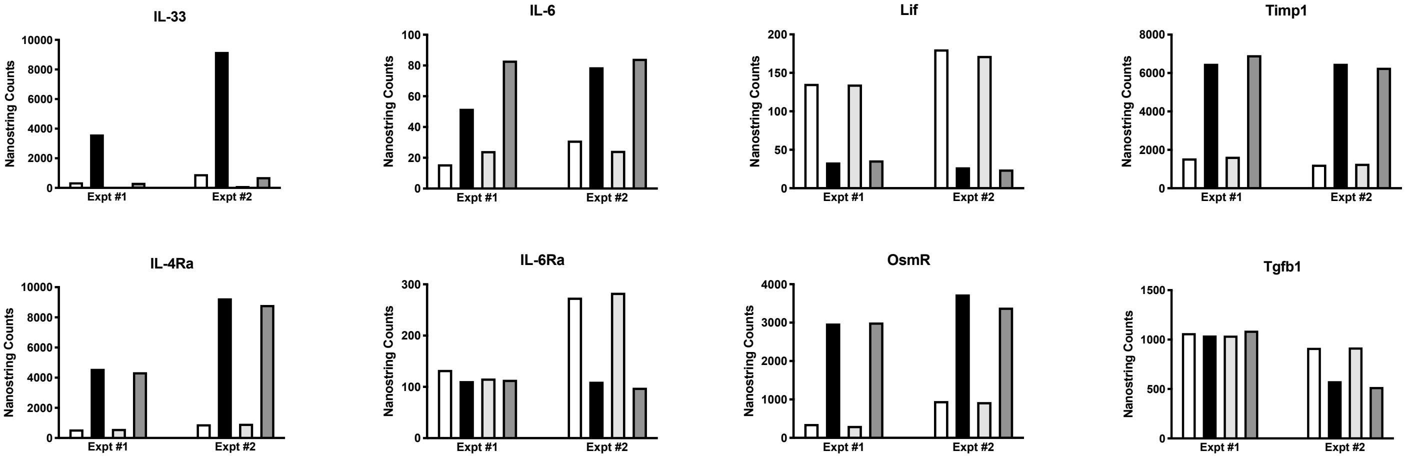

Supplemental Figure S5. $1x10^6$ C10 alveolar epithelial cells were transfected with SMARTpool ON-TARGETplus IL-33 siRNA (mIL-33 siRNA) or scrambled siRNA (SCRAM.) followed by stimulation with murine OSM (mOSM) for 24 hours, or remained untreated (Control). RNA was extracted using a PureLink RNA Mini Kit and mouse IL-33, IL-6, leukemia inhibitory factor (LIF), TIMP-1, IL-4 receptor alpha (IL-4Ra), IL-6 receptor alpha (IL-6Ra), OSM receptor beta (OsmR) and TGFbeta1 accessed by quantitative Nanostring Technologies.

Supplemental Figure S6

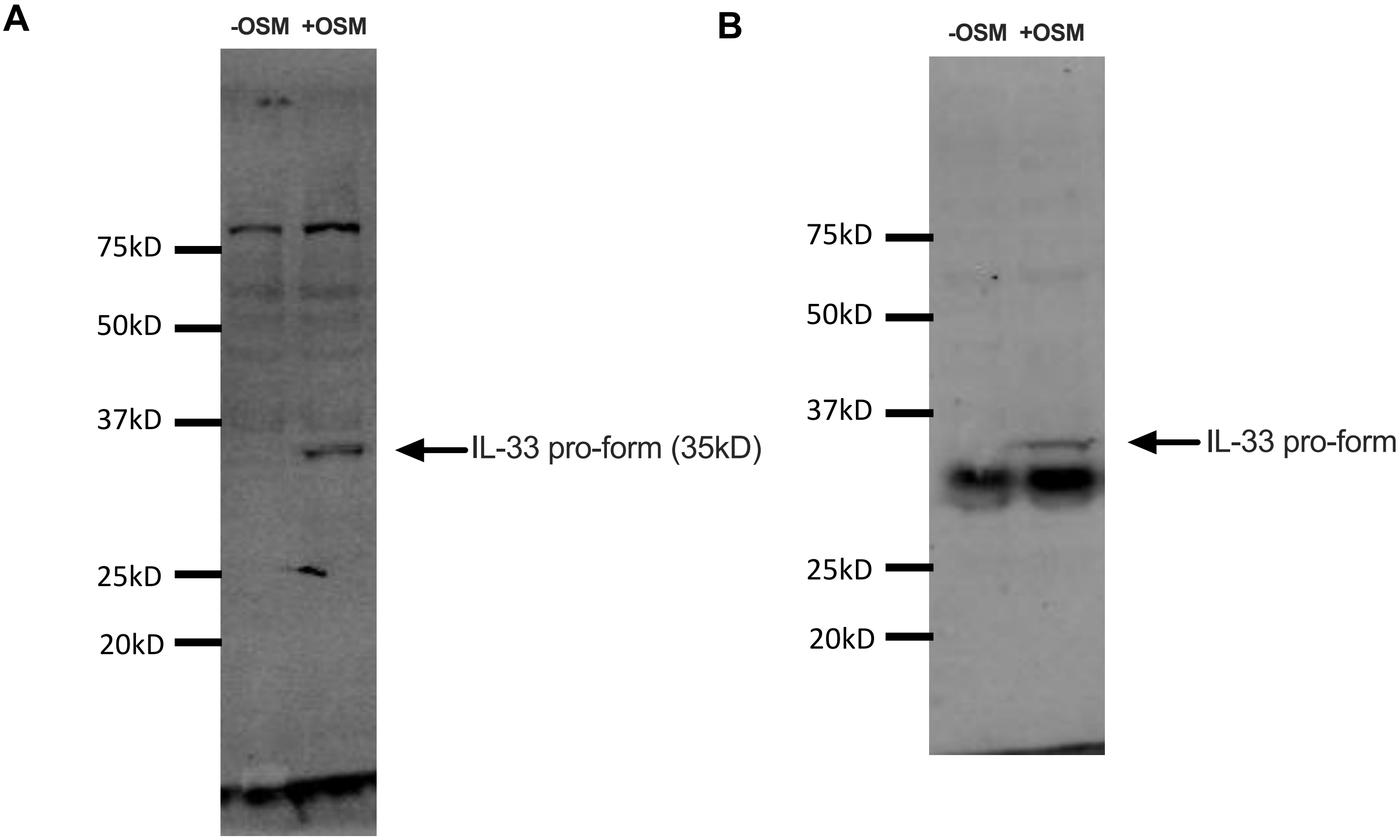

Supplemental Figure S6. Immunoblot detection of IL-33 in human A549 cells. Full length blots of IL-33 Western Blotting shown in Figure 9B using the Nessy-1 mouse monoclonal antibody (A) or when using similar whole cell lysates with the R&D goat polyclonal antibody (B). Position of IL-33 pro-form is denoted by the black arrow.

Supplemental Figure S7

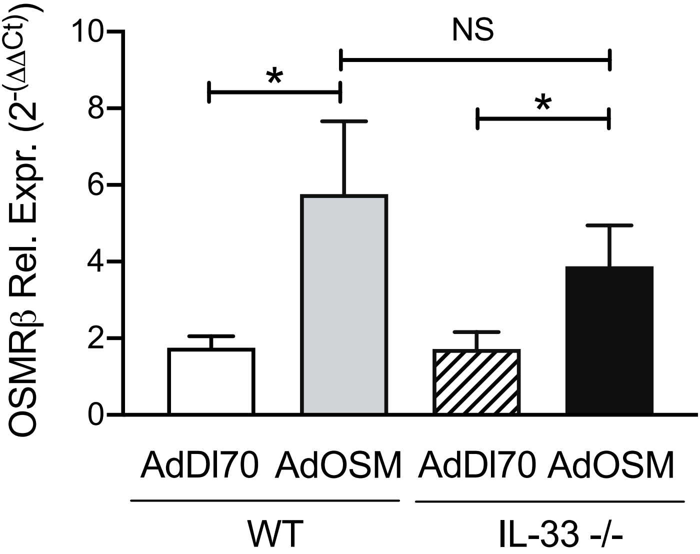

Supplemental Figure S7. AdOSM-induced OSMRb expression in C57Bl6 wildtype and IL-33-/- mice. C57Bl/6 wildtype (WT) or IL-33-/- mice were endotracheally administered AdDl70(control) or AdOSM and analyzed after 7 days for OSM receptor beta (OSMRβ) mRNA expression from whole lung by qPCR.
